# Soundless Trouble: Syringe Pump Malfunction and the Hypotension Threat

**DOI:** 10.7759/cureus.56996

**Published:** 2024-03-26

**Authors:** Jakkireddy Sravani, Chinmaya Panda, Mussavvir Agha, Swati Vijapurkar, Gade Sandeep

**Affiliations:** 1 Anaesthesiology, Critical Care, and Pain Medicine, All India Institute of Medical Sciences Raipur, Raipur, IND; 2 Anaesthesiology, All India Institute of Medical Sciences Raipur, Raipur, IND; 3 Emergency Medicine and Trauma, All India Institute of Medical Sciences Raipur, Raipur, IND

**Keywords:** driving pressure, occlusion limit, syringes, infusion pump, drug delivery

## Abstract

Drug infusion devices have become indispensable tools in ICU patient care, drug delivery, and operation rooms (OR) and for controlled fluid delivery. Syringe pump safety is paramount in healthcare and laboratory settings to ensure accurate medication delivery and prevent adverse events. Healthcare professionals must receive thorough training on syringe pump operation, including loading syringes, programming infusion rates, and responding to alarms. Using the correct syringe size and type is essential to prevent inaccuracies in drug/fluid delivery. Regular calibration and maintenance checks are necessary to ensure the accuracy and reliability of the syringe pumps. Two cases of refractory hypotension are reported here, which were resolved by careful inspection of the infusion pumps.

## Introduction

Drug infusion devices are an important part of drug delivery in intensive care unit (ICU) patient care as well as in the operation rooms (OR). Drugs are delivered via these electromechanical systems with moderate precision [1]. This marvel of invention has to be acknowledged. But this does not come without a few limitations. For a safe drug delivery, a calibrated infusion pump with a functional syringe and a pressure monitoring line without any leaks is required. Here we report two curious cases of refractory hypotension which were resolved by careful inspection of the infusion pumps. 

## Case presentation

Case 1

**Figure 1:**
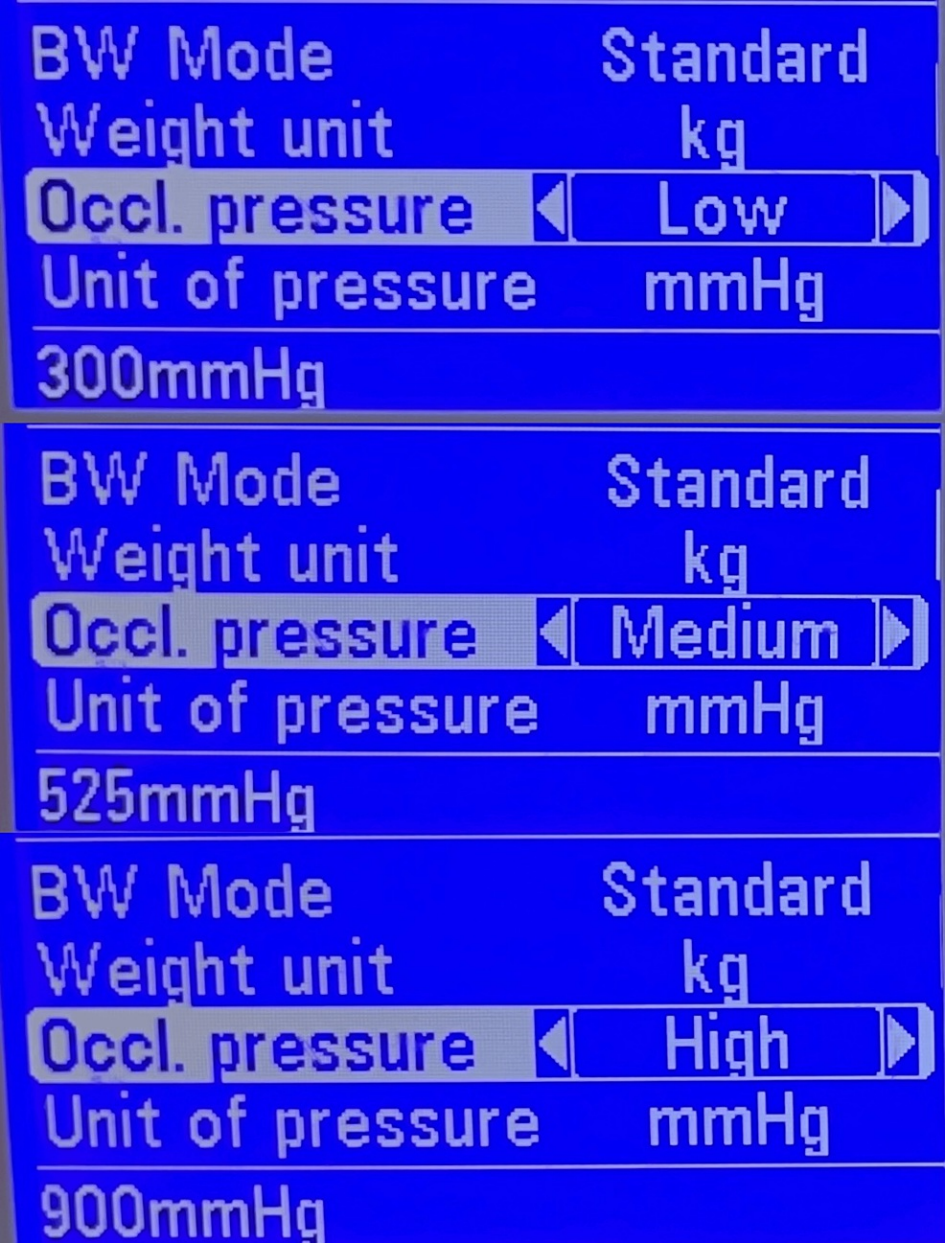
Range of occlusion pressure limits on the infusion pump. This is the display screen of the infusion pump, Mindray Benefusion SP3, showing body weight mode and unit of the patient. It shows the occlusion pressure levels: low is 300 mmHg; medium is 525 mmHg and high occlusion pressure is 900 mmHg. Alarm sounds when the occlusion pressure rises above the set level.

A 53-year-old female was admitted in our ICU with a diagnosis of urosepsis and septic shock. A triple lumen central venous catheter (CVC) was secured in the right internal jugular vein. Backflow was confirmed and present in all the three lumen. The patient required inotropic support in the form of noradrenaline and adrenaline which was administered via the CVC and titrated as per the blood pressure. Patient’s blood pressure was monitored via a radial arterial line. The infusion syringe pump (Benefusion SP3, Mindray, Shenzhen, China) was calibrated, fully charged and all the alarm limits were set at a minimum value. Patient’s blood pressure was fluctuating over a wide range of values from 90/50 mmHg to 200/140 mmHg and this wide fluctuation was seen over several seconds. Zeroing of the arterial line and square wave test were done to check for the dampening of the arterial system. There seemed to be no problem with the arterial line. On carefully observing the infusion pump, it revealed a high driving pressure of 500-600 mmHg, as seen in Figure 1, followed by a sudden release of the pressure to 60-70 mmHg which was corresponding with the fluctuations in the blood pressure. The release of the high driving pressure led to a bolus dose of the inotropes which caused an increase in the blood pressure. Subsequently there was a fall in blood pressure as the driving pressure in the infusion pump decreased to a value of 60-70 mmHg. Therefore an error in the infusion pump/syringe was established. All the infusion pumps were checked with the same syringe and it showed similar findings. Hence the particular type of syringe was changed and the problem was resolved. Unfortunately, the patient couldn’t survive and expired due to multiorgan failure three days later. 

Case 2

**Figure 2:**
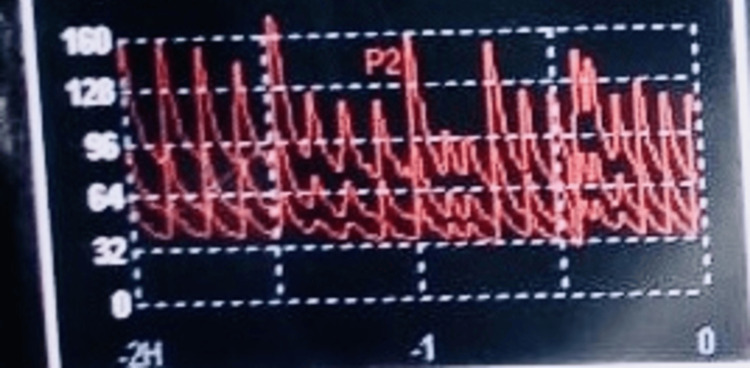
Trend showing the wide fluctuations in blood pressure (mmHg) Invasive arterial blood pressure waveform showing the wide fluctuations in blood pressure (mmHg)

A similar incident was observed in the operation theatre in a 27-year-old male patient undergoing mitral valve replacement. During weaning from the cardiopulmonary bypass, the patient required inotropic support with dobutamine and noradrenaline for the maintenance of the blood pressure. It was observed that following a brief period of optimum blood pressure, there was a sudden fall in blood pressure which did not improve despite increasing the rate of the infusions, leading to wide fluctuations in blood pressure, as seen in Figure 2. After ruling out all the other possible causes of hypotension, a careful inspection of the syringe pumps was done. It was observed that the infusion was being delivered in pulses after reaching a high driving pressure, rather than as a continuous infusion which led to periods of hypotension followed by a brief period of normotension/ hypertension. Syringes of that particular make were then replaced and the problem was resolved. 

## Discussion

The sheer scale of drug delivery that the infusion pumps have to cater to is immense. From 0.2-0.3 ml/hr of concentrated drugs to 100 ml/hr of antibiotic infusions, added to the fact that these drugs vary in viscosity and also the effect of gravity on infusions [2]. Infusion pumps can be of many types like gravity feed, in-line piston, peristaltic drive, and syringe pumps [1]. More commonly used in our setup are syringe pumps. These pumps function quite well and have an accuracy of ± 5% under laboratory conditions [3].

Syringe pumps work by a motor that drives the plunger linearly based on the set rate and the diameter of the syringe. The syringe diameter is sensed by the ratchet holding the syringe or has to be manually put in by specifying the make of the syringe. The pump starts to push the plunger of the syringe till a particular flow rate is reached. There is some time delay in starting the pump and actual drug delivery. Firstly, the pump has to overcome stiction (coefficient of static friction) between the plunger and the body of the syringe [4]. Larger syringes have a greater area of contact of the plunger to the syringe which prolongs the duration of their start time.

Syringe pumps have a comprehensive alarm system that detects any occlusion in the infusion line and alerts the provider. When flow is occluded in the line, the pressure will rise until it reaches the set occlusion pressure. Occlusion pressures can be set at three levels depending on the make of the syringe pump, this setting is usually displayed on the electronic pump screen. Time to reach this occlusion pressure depends on various factors. 

The larger the syringe or slower the rate, the more time it takes to reach the set pressure [5-7]. More compliance in the infusion line can also be a cause of a delay in the alarm [8]. Increasing the length of the infusion lines can critically prolong the alarm [9]. Since the fluid in the infusion line is compressible, when pressure increases due to an occlusion, its volume decreases. The pump interprets this as drug delivery, but as volume decreases, the pressure increases. When this pressure surpasses the predetermined occlusion pressure, the pump stops the motor and sounds the alarm. At this point the fluid decompresses and the volume in the syringe increases pushing back the plunger.

In our case, the friction of the syringe plunger was more due to some manufacturing defect. This caused the pump to increase the pressure to reach the set flow rate but as the pressure increased, the plunger gave way only at high pressure which was lower than the set occlusion pressure. When the plunger moved forward, the pressure decreased subsequently and the motor continued to push. This caused the drug to be administered in pulses and not as an infusion and there was no alarm. 

With this experience, we suggest that 1) alarms of the infusion pumps should be set properly; 2) the length of infusion lines must be decreased as much as possible; 3) for multiple infusions connected to a single line, the flow rates of the syringes must be set at comparable rates; 4) smaller syringes should be used for low infusion rates and concentrated drugs [10]; 5) standard care should be taken in preparing the syringes and drugs with minimum interference when preparation is done and the selection of the line to connect the infusion should be proactively done with the immediate setting of alarms of the connected pumps [11]; 6) decrease alarm tolerance as much as possible by proper training and motivation.

## Conclusions

Hence comprehensive education and training for ICU staff, doctors and OR technicians on the proper functionality of infusion pump systems, coupled with the implementation of a sequential checklist before initiating any infusion, are essential measures in mitigating mechanical errors and minimizing adverse effects. These proactive steps not only enhance patient safety but also promote the efficient and accurate delivery of medications, underscoring their critical role in the realm of modern healthcare.
